# Spontaneous 8bp Deletion in *Nbeal2* Recapitulates the Gray Platelet Syndrome in Mice

**DOI:** 10.1371/journal.pone.0150852

**Published:** 2016-03-07

**Authors:** Kärt Tomberg, Rami Khoriaty, Randal J. Westrick, Heather E. Fairfield, Laura G. Reinholdt, Gary L. Brodsky, Pavel Davizon-Castillo, David Ginsburg, Jorge Di Paola

**Affiliations:** 1 Department of Human Genetics, University of Michigan, Ann Arbor, Michigan, United States of America; 2 Department of Internal Medicine, University of Michigan, Ann Arbor, Michigan, United States of America; 3 Department of Biological Sciences, Oakland University, Rochester, Michigan, United States of America; 4 The Jackson Laboratory, Bar Harbor, Maine, United States of America; 5 Department of Pediatrics, University of Colorado Denver, Aurora, Colorado, United States of America; 6 Life Sciences Institute, University of Michigan, Ann Arbor, Michigan, United States of America; 7 Howard Hughes Medical Institute, University of Michigan, Ann Arbor, Michigan, United States of America; 8 Human Medical Genetics and Genomics Program, University of Colorado Denver, Aurora, Colorado, United States of America; Medical Faculty, Ludwig Maximilians University Munich, GERMANY

## Abstract

During the analysis of a whole genome ENU mutagenesis screen for thrombosis modifiers, a spontaneous 8 base pair (bp) deletion causing a frameshift in exon 27 of the *Nbeal2* gene was identified. Though initially considered as a plausible thrombosis modifier, this *Nbeal2* mutation failed to suppress the synthetic lethal thrombosis on which the original ENU screen was based. Mutations in *NBEAL2* cause Gray Platelet Syndrome (GPS), an autosomal recessive bleeding disorder characterized by macrothrombocytopenia and gray-appearing platelets due to lack of platelet alpha granules. Mice homozygous for the *Nbeal2* 8 bp deletion (*Nbeal2*^*gps/gps*^) exhibit a phenotype similar to human GPS, with significantly reduced platelet counts compared to littermate controls (p = 1.63 x 10^−7^). *Nbeal2*^*gps/gps*^ mice also have markedly reduced numbers of platelet alpha granules and an increased level of emperipolesis, consistent with previously characterized mice carrying targeted *Nbeal2* null alleles. These findings confirm previous reports, provide an additional mouse model for GPS, and highlight the potentially confounding effect of background spontaneous mutation events in well-characterized mouse strains.

## Introduction

The laboratory mouse has been used extensively as a model organism, with multiple inbred mouse strains routinely available from a number of suppliers. These inbred strains have been extensively characterized and the genome of more than 20 have been sequenced [1, 2]. Whole genome sequencing in humans has demonstrated that in addition to approximately 75 *de novo* single nucleotide variants (SNVs) [3], each human genome carries on average 6–12 new insertions and deletions or 'INDELs' (1–50 bp) and occasional copy number and complex structural variants [4, 5]. Mice have been shown to exhibit comparable mutation rates [6] and therefore elaborate breeding schemes are necessary in large mouse facilities to maintain genetically stable mouse strains [7]. However, identification of the occasional *de novo* deleterious variants in mice has resulted in useful models for phenotypic studies [8–11]. Forward genetic screens can be performed taking advantage of such spontaneous mutations, but given the low *de novo* mutation rate, N-ethyl-N-nitrosourea (ENU) is typically applied to markedly increase the density of random mutations [12, 13]. ENU induces on average 1 mutation per every 700,000 bp, which results in >50 fold increase compared to spontaneous mutation rates seen in mice [14, 15].

*NBEAL2* encodes neurobeachin-like-2, a BEACH domain containing protein, with a proposed role in vesicular trafficking and granule development [16]. Mutations in *NBEAL2* were recently shown to be the cause of the autosomal recessive form of Gray Platelet Syndrome (GPS) [17–19]. GPS is a rare bleeding disorder characterized by macrothrombocytopenia and gray-appearing platelets due to lack of platelet alpha granules [20]. Mice with targeted deletion of *Nbeal2* [21–23] exhibit thrombocytopenia, deficiency in platelet alpha granules, a higher than normal mean platelet volume, splenomegaly, impaired platelet aggregation and adhesion, and a mild bleeding tendency, all consistent with the human phenotype [20, 24].

During the analysis of a whole genome ENU mutagenesis screen for thrombosis modifiers, we identified a spontaneous 8 bp deletion causing a frameshift in exon 27 of the *Nbeal2* gene. Analysis of the associated mouse pedigree demonstrated that this mutation arose within the Jackson laboratory 129S1/SvImJ mouse colony and not from the ENU screen.

## Materials and Methods

### Animal procedures

Animal husbandry in this study was carried out according to the Principles of Laboratory and Animal Care established by the National Society for Medical Research. The University of Michigan’s University Committee on Use and Care of Animals (UCUCA) has approved the protocol number 05191 and the University of Colorado Institutional Animal Care and Use Committee approved the protocol 96114. The care and maintenance of animals was closely supervised by University of Michigan ULAM personnel or University of Colorado Institutional Animal Care and Use Committee (IACUC) and animals were housed in their facilities. ULAM/IACUC also provided expert veterinary advice and assistance when necessary and cages were monitored closely by our laboratory personnel as well as university veterinary staff. To minimize discomfort and unnecessary suffering of experimental mice, analgesics were administered for all procedures involving significant discomfort. Blood samples were obtained from the retro-orbital plexus of anesthetized animals achieved with isoflurane inhalation. Mice were euthanized for collection of tissues for histologic, biochemical, and genetic analysis. The UCUCA Endstage Illness and Humane Endpoint Guidelines were also closely followed and animals euthanized accordingly by carbon dioxide overdose or exsanguination under anesthesia.

*F5*^*L/L*^ (*F5*^*tm2Dgi*^/J stock number 004080) mice were previously generated [25], *Tfpi* deficient mice (*Tfpi*^*tm1Gjb*^) were a generous gift of Dr. George Broze [26], and *Nbeal2*^*tm1Lex/tm1Lex*^ mice with targeted deletion of the *Nbeal2* gene were previously generated from cryopreserved spermatozoa obtained from the Mutant Mouse Regional Resource Center at the University of California, Davis [22]. *Nbeal2* allele carrying the spontaneous 8bp deletion described in Results will be referred throughout the text as *Nbeal2*^*gps*^. Two cohorts of *Nbeal2*^*gps*^ mice were analyzed. Set 1 refers to *Nbeal2*^*gps*^ mice intercrossed after 2 backcrosses to C57BL/6J mice (stock number 000664), while set 2 mice were intercrossed after 7 backcrosses to C57BL/6J.

### Whole exome sequencing of thrombosis suppressor line

Genomic DNA (gDNA) was extracted from mouse tail biopsies using the Gentra Puregene Tissue Kit (Qiagen) according to manufacturer’s instructions. Exonic DNA was captured with either SureSelect Mouse All Exon (Agilent) or SeqCap EZ Mouse Exome Design (NimbleGen) kits and 100 bp paired-end sequencing was performed on the Illumina HiSeq 2000 platform at the University of Michigan's DNA Sequencing Core. All generated fastq files have been deposited to the NCBI Sequence Read Archive (Project accession number #SRP063933). Detailed overview of the variant calling pipeline and filtration is available online as a GitHub repository [27]. In short, reads were aligned with the Burrows-Wheeler Aligner [28] to the *Mus Musculus* GRCm38 reference genome, duplicates were removed using Picard [29], and variants across all samples were simultaneously called and filtered with GATK [30]. Variants were annotated using Annovar software [31] with Refseq annotation. Variants between C57BL/6J and 129S1/SvImJ, as well as unannotated variants within our mouse cohort present in more than one independent line, were removed from the ENU candidate list. All unique heterozygous variants present in multiple mice within the suppressor line *MF5L6* with a minimum of 6X coverage were considered as potential candidates and further validated using Sanger sequencing.

### 129S1/SvImJ *de novo* mutation analysis

Exome analysis was performed for the parents (F63pF64) and a female sibling (F63pF65) of the 129S1/SvImJ individual sequenced for the Sanger Mouse Genomes Sequencing project [1]. Exome sequencing and variant calling was performed as previously described [32]. Approximately 95% of all variants (SNV and INDELs) in each of the 3 samples were also found in dbSNP, and an additional ~5600 variants that were common between the three exomes were also found in whole genome variant data from the Sanger Mouse Genomes project. There were 92 variants unique to the 129S1/SvImJ female sibling sample, in that they were not found in variant calls from either parental exome. Out of the 92 “unique” called variants, manual analysis of the alignment files in all samples and Sanger sequencing of PCR products revealed that 91 were true variants with false negative calls in one of the parent samples and one variant was a false positive call.

### Genotyping *Nbeal2*^*gps*^ allele

The *Nbeal2*^*gps*^ allele was detected using two three-primer PCR assays (S1 Fig) with common forward (5'AAGGCAGGAAGACGTCAGAA, primer F) and reverse (5'GACCTCAGTGTCCGCCTAGA, primer R) primers. In the first PCR based genotyping design, the third primer (5'AC**|GTCTGGCT|**GTCCGTAGAT, primer WT) is located over the undeleted 8 bp to detect the presence of the wildtype allele. This PCR reaction results in two products (413 bp, 235 bp). In the second PCR design the third primer (5'AACGAC|GTCCGTAGATGAGG, primer DEL) spans the 8 bp deletion to detect the presence of the deletion allele. This reaction also produces two products (405 bp, 227 bp). PCR was performed using GoTaq Green Master Mix (Promega) and the products visualized on a 2% agarose gel. Selected genotyping results were further confirmed by Sanger sequencing.

### Estimation of differential allelic expression

Liver, lung, and bone marrow tissue samples were collected in RNAlater (Ambion) from a *Nbeal2*^*gps/+*^ mouse. Total RNA was extracted using an RNeasy Mini Kit (Qiagen) and converted to coding DNA (cDNA) using SuperScript III One-Step RT-PCR (Invitrogen) following the manufacturer’s instructions. gDNA was prepared from a tail biopsy. Forward and reverse genotyping primers (primer F and primer R) were used to amplify the *Nbeal2* deletion region from gDNA and the cDNAs from liver and lung. PCR products were extracted from agarose gels using a QIAquick Gel Purification Kit (Qiagen) and submitted for Sanger sequencing. The differential allelic expression was estimated from the ratio between the wildtype and *Nbeal2*^*gps*^ sequence peak areas in cDNA samples compared to gDNA using Phred software [33]. This ratio was calculated for multiple positions within the PCR product where the wildtype and *Nbeal2*^*gps*^ alleles contain a different nucleotide.

### Western blot

Murine whole blood was collected via the inferior vena cava into acid/citrate/dextrose. Platelet-rich plasma (PRP) was obtained by centrifugation at 200 g for 5 min. Washed platelets were pelleted from PRP by centrifugation at 1,000 g for 2 min in the presence of prostacyclin PGI1 (0.1 μM) and resuspended in modified Tyrode’s buffer (137 mM NaCl, 0.3 mM Na_2_HPO_4_, 2 mM KCl, 12 mM NaHCO_3_, 5 mM HEPES, 5 mM glucose) [34]. Total protein was harvested from washed platelets using cell lysis buffer containing 1% Triton X-100 and protease inhibitors (mini complete tablets, Roche). Protein concentration was measured with Protein Dye Reagent (BioRad), and 30 μg of total protein was separated in duplicate lanes of a 4–15% Mini-Protean TGX gel (BioRad). Protein was transferred to nitrocellulose and probed with a rabbit monoclonal antibody against NBEAL2 (ab187162, Abcam) or a rabbit polyclonal antibody against beta actin (ab8227, Abcam), followed by an HRP-linked goat anti-rabbit secondary antibody (Pierce). Detection was performed with ECL Lightning Plus (Perkin Elmer).

### Complete blood counts

Twenty-five microliters of blood were collected from the retro-orbital sinus of 5–6 week old mice from set 1. Blood was anticoagulated with 4% sodium citrate (Sigma-Aldrich) and diluted 10x in PBS (phosphate-buffered saline, Gibco) supplemented with 5% bovine serum albumin (Sigma-Aldrich). Complete blood counts (CBC) were performed on the ADVIA 2120 Hematology System (Siemens) according to manufacturer’s instructions while being blinded to the genotype of the mouse from which the sample was obtained. Additional blood was collected from >20 week old females from set 2 using heparinized capillary tubes and anticoagulated using EDTA containing tubes (BD microtainer). CBC were performed on the Hemavet 950FS system. All data were analyzed and visualized using the stats and beeswarm packages in R software [35].

### Flow cytometry

Absolute neutrophil counts (ANC) were measured by flow cytometry as previously described [36]. Briefly, 50 μl of anticoagulated whole blood was added to Trucount tubes (BD Biosciences) and processed according to the manufacturer’s protocol. Samples were incubated at room temperature in the dark for 15 minutes with rat anti-mouse FITC-conjugated Ly-6G clone 1A8 (Molecular Probes) and rat anti-mouse CD45 PE/Cy7 antibodies. This incubation was followed by the addition of 450 μl of red blood cell lysis buffer (eBioscience). Samples were incubated for 30 minutes in the dark at room temperature prior to data acquision using a Gallios 561 flow cytometer (Beckman Coulter). Data were acquired at medium flow rate for 2 minutes. The neutrophil population was defined as CD45/Ly-6G positive events. The bead population was clearly visualized as different from the neutrophil population. The ANC was calculated according to the formula provided by the manufacturer: ANC (cells/ μl) = (CD45 and Ly-6G positive events/ Trucount beads)x(# beads per test/test volume).

### Peripheral blood and bone marrow analysis

Peripheral blood smears were prepared from 9 mice of each genotype and Wright-Giemsa stained using the HealthCare PROTOCOL Hema 3 kit according to the manufacturer’s instructions (Fisher Scientific). For each sample, the intensity of platelet staining and platelet granularity were categorized into three levels (light, intermediate or dark) by one of the authors (RK) blinded to the genotype of the mouse from which the sample was obtained. Representative images from the blood smears were taken using a Leica DMLB microscope at 1000x magnification. Bone marrow sections as well as bone marrow cytology slides were prepared by the Unit for Laboratory Animal Medicine histology core. Histopathologic evaluation was performed by an investigator blinded to the genotypes of the evaluated mice.

### Transmission electron microscopy

Blood from one *Nbeal2*^+/+^ and one *Nbeal2*^*gps/gps*^ mouse from set 1 was collected by retro-orbital puncture and fixed in 4% glutaraldehyde as previously described [37]. Fixed samples were further prepared by the University of Michigan's Microscopy and Image Analysis Core for platelet transmission electron microscopy. Platelet sections were examined on a JEOL JEM-1400Plus transmission electron microscope at two different magnifications (5000x, 40000x). Platelet area was measured from images at 5000x magnification using ImageJ software [38] for 100 platelets from 5 different fields for each genotype. The latter analysis was performed with the observer blinded to the genotypes of the platelets.

### Statistical analysis

A non-parametric Wilcoxon test was used to estimate significance in the CBC measured values, platelet area, the assigned platelet staining intensity values of the Wright-Giemsa stained blood smears, and the difference in the level of emperipolesis in bone marrow slides between *Nbeal2*^*gps/gps*^ and wildtype mice. A chi-square test was applied to estimate deviations from expected Mendelian proportions in *Nbeal2* mouse crosses. All statistical analyses were performed using the stats package in R software [35].

## Results

### *De novo* frameshift mutation in *Nbeal2* identified by whole exome sequencing

**Fig 1:**
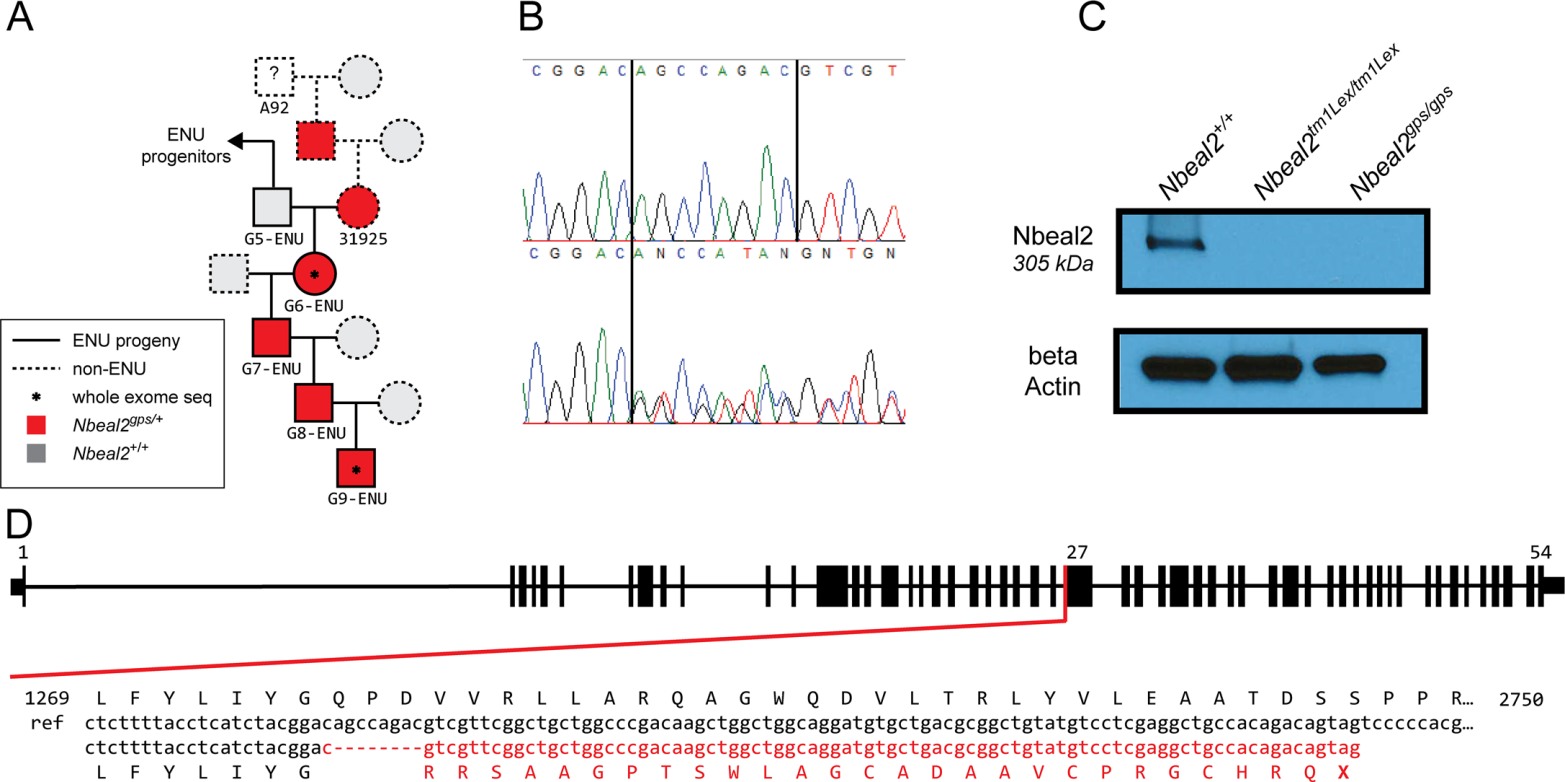
*De novo* 8 bp deletion in the *Nbeal2* gene. The whole exome sequenced G6-ENU mouse inherited the *Nbeal2* deletion from a non-ENU parent 31925 (A). Sanger sequencing validates the heterozygous frameshift mutation in the suppressor pedigree (B). Western blot analysis of washed mouse platelets show a band at the expected size for NBEAL2 (~305kDa) in wildtype mice. This band is missing in *Nbeal2*^*tm1Lex/tm1Lex*^ mice as well as mice homozygous for the *Nbeal2*^*gps*^ allele (C). Schematic overview of the *Nbeal2* gene, the location of the deletion and the expected frameshift (D).

We performed a sensitized dominant ENU screen designed to identify suppressor mutations for a synthetic lethal thrombosis phenotype (*F5*^L/L^ *Tfpi*^+/-^) in C57BL/6J mice [39]. In order to map the ENU induced mutations, outcrosses were performed between the mutagenized mice and *F5*^L/L^ mice [25] bred >12 generations to the 129S1/SvImJ genetic background. Within a suppressor line, all ENU induced mutations should segregate randomly to the next generation except the suppressor mutation, which is expected to be present in all *F5*^L/L^ *Tfpi*^+/-^ mice. Whole exome sequencing was applied to 4 mice from one of the suppressor lines (*MF5L*6, S2 Fig) and variants shared between the 4 mice were investigated as candidate suppressor mutations. A total of 215 unique exonic heterozygous SNVs and 8 heterozygous INDELs were identified in the 4 exomes from a total of 76,950 initially called variants. Twelve of the SNVs and one of the INDELs were present in more than one sequenced mouse (Table 1), while no variant was present in all 4 mice with the exception of variants closely linked to the *Tfpi*^-^ locus. The only shared INDEL (between G6-ENU and G9-ENU, Fig 1A) was an 8 bp deletion (AGCCAGAC) in the 27th exon of *Nbeal2*, confirmed by Sanger sequencing (Fig 1B). This allele will be denoted *Nbeal2*^*gps*^. Genotyping additional members of the pedigree demonstrated absence of this deletion allele in generation 5 (G5) ENU mutagenized progeny exhibiting the suppressor phenotype (Fig 1A). Instead, the G6-ENU mouse inherited the deletion from its non-ENU parent and would have been missed in our mouse cohort if it had not been coincidentally shared by the whole exome sequenced G9-ENU mouse. Absence of the *Nbeal2*^*gps*^ allele in the first five generations of ENU pedigree excludes *Nbeal2*^*gps*^ as the original suppressor mutation. Additionally, *Nbeal2*^*gps*^ failed to segregate with the suppressor phenotype in later generations (S2 Fig). Further genotyping identified a cohort of 129S1/SvImJ mice purchased from the Jackson Laboratory as the likely source of the deletion variant (S3 Fig).

**Table 1:** Overview of the exonic variants called from whole exome sequencing in 4 mice from the *MF5L*6 pedigree.

**TYPE**	**All SNVs**	**Unique SNVs**	**In > 1 mouse**
nonsense	87	3	1
nonsynonymous	11,854	66	6
synonymous	21,130	27	2
splice	90	2	0
exonic	34,288	117	3
TOTAL	67,449	215	12
**TYPE**	**All INDELs**	**Unique INDELs**	**In > 1 mouse**
frameshift	344	3	1 (*Nbeal2*)
nonframeshift	460	0	0
splice	71	1	0
exonic	8626	4	0
TOTAL	9501	8	1

Details available at github.com/tombergk/NBEAL2

### The *Nbeal2*^*gps*^ allele is not segregating in 129S1/SvImJ stock

To minimize cumulative genetic drift, the 129S1/SvImJ colony at The Jackson Laboratory is maintained under a Genetic Stability Program (GSP) [7]. In this scheme, foundation breeding colonies are maintained with cryopreserved embryos that are descendants of a single, founder breeder pair. To trace the origin of the *Nbeal2*^*gps*^ allele, six archived samples from the 129S1/SvImJ (JR# 002448) colony at The Jackson Laboratory were genotyped, including the 129S1/SvImJ founder pair, “Adam and Eve” (F60) as well as archived samples from before (F56, F59) and after (F61, F63) implementation of the GSP program. The *Nbeal2* deletion was not found in any of these samples (S3 Fig). Exome sequencing data from two additional 129S1/SvImJ samples (F63pF67) [32], whole genome sequencing data from the Sanger Mouse Genomes Project, F63pF65 [1], and exome sequencing data from a female sibling, as well as the dam and sire (F63pF64) of the 129S1/SvImJ individual sequenced by the Sanger Mouse Genomes project identified only wildtype *Nbeal2* (S3 Fig). Mice with the *Nbeal2*^*gps*^ allele were purchased after the implementation of GSP. Since neither Adam nor Eve were carriers, the deletion must have arisen later in the colony but is no longer segregating in the 129S1/SvImJ stock at The Jackson Laboratory.

### An 8 bp deletion results in a frameshift mutation in *Nbeal2*

The identified 8bp deletion in *Nbeal2* is expected to cause a frameshift that introduces an early stop codon 28 amino acids downstream of the deletion site (Fig 1D). The expression level of *Nbeal2*^*gps*^ mRNA from bone marrow, liver, and lung tissues was assessed by RT-PCR and Sanger sequencing. Although *Nbeal2*^*gps*^ mRNA could be detected by RT-PCR, the level was ~64% lower in bone marrow, ~73% lower in liver, and ~59% lower in lung compared to the wildtype allele (S4 Fig). These results are consistent with nonsense-mediated decay [40]. In addition, no band was detected at the expected size (~305kDa) by western blot analysis of washed platelets obtained from *Nbeal2*^*gps/gps*^ mice (Fig 1C) and no truncated protein was observed with an N-terminal antibody (S5 Fig).

### *Nbeal2*^*gps/gps*^ mice are viable and fertile

A mouse carrying the *Nbeal2* deletion allele (S2 Fig) was outbred from the ENU suppressor line for two generations to remove the *F5*^L^ and *Tfpi*^*-*^ mutant alleles, as well as the majority of residual, unlinked ENU induced variants. Mice carrying one (*Nbeal2*^*gps/+*^) or two deletion alleles (*Nbeal2*^*gps/gps*^) were viable, fertile and had no apparent phenotype by visual inspection. No significant deviation from the expected Mendelian distribution was observed in the progeny when crossing the *Nbeal2*^*gps/+*^ mice to C57BL/6J wildtype mice or in the progeny from the *Nbeal2*^*gps/+*^ intercross (Table 2).

**Table 2:** Expected and observed number of progeny in *Nbeal2*^*gps/+*^ crosses.

Cross	*Nbeal2*^*+/+*^	*Nbeal2*^*gps/+*^	*Nbeal2*^*gps/gps*^	p-value*
***Nbeal2***^***gps/+***^ **x *Nbeal2***^***+/+***^	46% (37)	54% (44)	-	0.4367
expected	50%	50%	-	
***Nbeal2***^***gps/+***^ **x *Nbeal2***^***gps/+***^	24% (23)	47% (44)	29% (27)	0.6965
expected	25%	50%	25%	

*A chi-square test was applied to estimate deviations from expected Mendelian proportions.

Number of mice genotyped available in parentheses.

### *Nbeal2*^*gps/gps*^ mice exhibit thrombocytopenia and neutropenia

**Fig 2:**
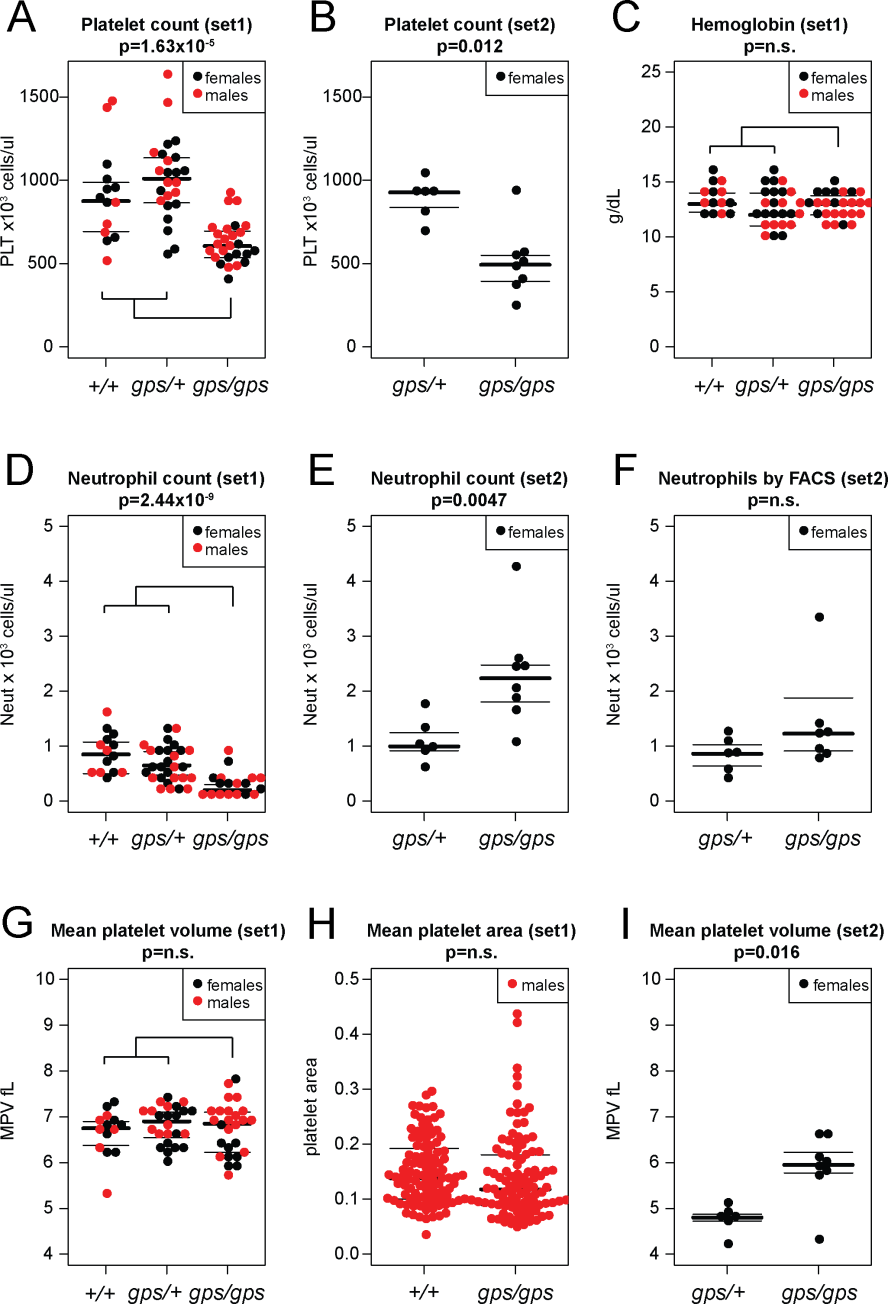
Comparison of CBCs. Platelet counts are lower in *Nbeal2*^*gps/gps*^ mice compared to control mice in both set 1 (A) and set 2 (B) mice while hemoglobin levels are similar between the two groups (C). *Nbeal2*^*gps/gps*^ mice from set 1 exhibit significant neutropenia (D), which is not observed in set 2 by CBC (E) or flow cytometry (F). Mean platelet volume (G) and area (H) do not differ in set 1 mice, but show an increase in size for *Nbeal2*^*gps/gps*^ mice in set 2 (I).

Complete blood counts (CBC) were performed on 24 *Nbeal2*^*gps/+*^, 26 *Nbeal2*^*gps/gps*^ mice, and 14 wildtype littermate controls from set 1. No significant differences were observed between *Nbeal2*^*gps/+*^ and *Nbeal2*^+/+^ mice in any of the measured parameters (Table 3) and those genotypes were subsequently grouped together as controls for comparison to *Nbeal2*^*gps/gps*^ mice. Platelet counts of *Nbeal2*^*gps/gps*^ mice were significantly reduced compared to control mice (623 vs 968 x 10^3^ cells/μl, p = 1.63 x 10^−7^) as was the absolute neutrophil count (0.27 vs 0.77 x 10^3^ cells/μl, p = 2.44 x 10^−9^) (Table 3, Fig 2). All other CBC parameters, including mean platelet volume, were indistinguishable between *Nbeal2*^*gps/gps*^ and control mice (Table 3). In addition, no difference was observed in mean platelet area quantitated in electron microscopy images. However, in CBCs obtained from a second cohort of 6 *Nbeal2*^*gps/+*^ and 8 *Nbeal2*^*gps/gps*^ females, both neutrophil counts (p = 0.0047) and mean platelet volume (p = 0.016) were higher in the *Nbeal2*^*gps/gps*^ mice compared to littermate controls (Fig 2). Additional analysis of neutrophil counts for the set 2 mice by flow cytometry showed no significant differences.

**Table 3:** CBC mean values and standard deviations by each phenotype in set 1.

abbr.	*Nbeal2*^*+/+*^ mean ± sd	*Nbeal2*^*gps/+*^ mean ± sd	p-value	*Nbeal2*^*+/+*, *gps/+*^ mean ± sd	*Nbeal2*^*gps/gps*^ mean ± sd	p-value*
WBC	6.80 ± 1.46	7.23 ± 2.08	0.75	7.07 ± 1.86	7.04 ± 2.34	0.56
RBC	9.45 ± 0.85	8.96 ± 1.08	0.17	9.14 ± 1.02	8.91 ± 0.68	0.46
HGB	13.43 ± 1.28	12.58 ± 1.74	0.12	12.89 ± 1.62	12.77 ± 1.11	0.79
HCT	4.89 ± 0.39	4.66 ± 0.59	0.23	4.74 ± 0.53	4.68 ± 0.34	0.65
MCV	51.88 ± 2.05	52.06 ± 1.72	0.81	51.99 ± 1.82	52.66 ± 1.63	0.13
MCH	14.19 ± 0.48	14.16 ± 0.70	0.84	14.17 ± 0.62	14.47 ± 0.72	0.18
MCHC	27.41 ± 0.95	27.19 ± 1.00	0.62	27.27 ± 0.97	27.48 ± 1.04	0.48
CHCM	28.28 ± 2.02	28.20 ± 2.08	0.95	28.23 ± 2.03	27.10 ± 1.91	0.11
CH	14.67 ± 0.88	14.68 ± 0.83	0.84	14.67 ± 0.84	14.27 ± 1.02	0.09
RDW	15.94 ± 1.93	16.25 ± 1.85	0.40	16.13 ± 1.86	16.16 ± 1.32	0.50
HDW	1.66 ± 0.21	1.61 ± 0.14	0.73	1.63 ± 0.17	1.52 ± 0.09	4.44x10^-3^
PLT	906.4 ± 280.6	1004.6 ± 247.6	0.15	968.4 ± 260.9	622.7 ± 128.6	**1.63x10**^**-7**^
MPV	6.64 ± 0.51	6.80 ± 0.40	0.36	6.74 ± 0.45	6.72 ± 0.57	0.78
Neut	0.86 ± 0.36	0.73 ± 0.29	0.27	0.77 ± 0.32	0.27 ± 0.19	**2.44x10**^**-9**^
Lymph	5.05 ± 1.17	5.57 ± 2.10	0.35	5.38 ± 1.81	5.97 ± 2.46	0.56

WBC (White Blood Cell count), RBC (Red Blood Cell count), HGB (Hemoglobin concentration), HCT (Hematocrit), MCV (Mean Corpuscular Volume), MCH (Mean Corpuscular Hemoglobin), MCHC (Mean Corpuscular Hemoglobin Concentration), CHCM (Corpuscular Hemoglobin Concentration Mean), CH (Cellular Hemoglobin Content), RDW (Red Cell Volume Distribution Width), HDW (Hemoglobin Concentration Distribution Width), PLT (Platelet count), MPV (Mean Platelet Volume), Neut (Neutrophil cell count), Lymph (Lymphocyte cell count).

* Significant p-values after Bonferroni correction (p-value ≤ 0.0033) for multiple testing are highlighted in bold font

### *Nbeal2*^*gps/gps*^ platelets are deficient in alpha granules

**Fig 3:**
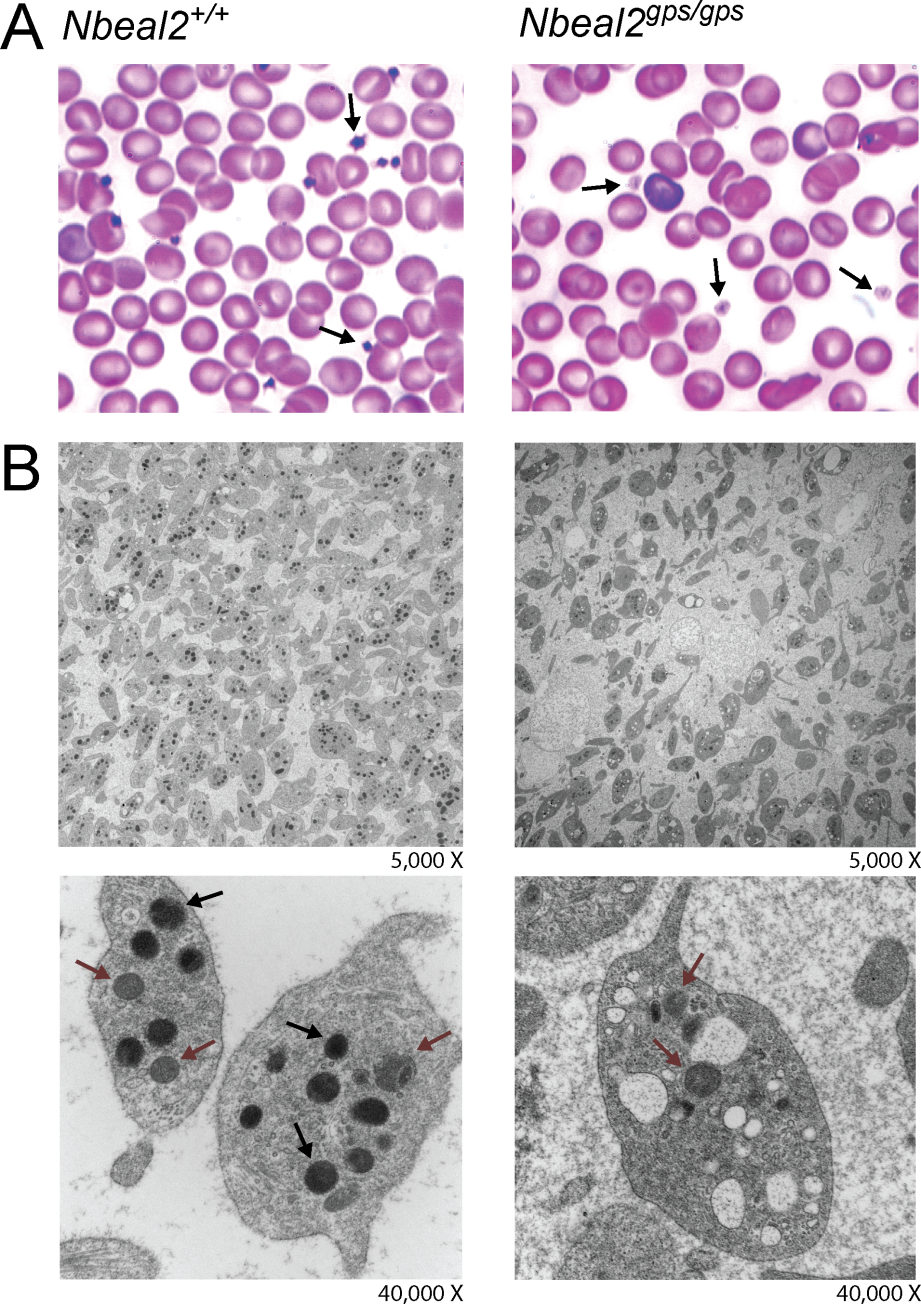
Deficiency in platelet alpha granules. *Nbeal2*^*gps/gps*^ platelets appear pale compared to wildtype (black arrows, A). Transmission electron microscopy (TEM) images show dark alpha granules in wildtype platelets (black arrows), which are missing in *Nbeal2*^*gps/gps*^ platelets. Red arrows indicate mitochondria (B).

The intensity of platelet staining with Wright-Giemsa dye was indistinguishable between *Nbeal2*^*gps/+*^ and wildtype mice (p-value = 0.298), but significantly reduced in *Nbeal2*^*gps/gps*^ mice (Fig 3A, Table 4, p-value = 1.9x10^-4^) consistent with a reduction in platelet alpha granules [20, 24]. Transmission electron microscopy also displayed a marked reduction of alpha granules in *Nbeal2*^*gps/gps*^ mouse compared to wildtype control (Fig 3B), consistent with the human GPS phenotype [20, 24].

**Table 4:** Intensity of platelet staining and frequency of emperipolesis events in bone marrow megakaryocytes.

**Intensity of platelet staining (n = 9)**					
**genotypes**	**light (1)**	**medium (2)**	**dark (3)**	**average**	**p-value**
*Nbeal2*^*+/+*^	0	1	8	2.89	
*Nbeal2*^*gps/+*^	0	3	6	2.67	0.298
*Nbeal2*^*gps/gps*^	7	2	0	1.22	0.0001898
Number of emperipolesis events (n = 3)*					
**genotypes**	**0**	**1**	**≥2**	**average**	**p-value**
*Nbeal2*^*+/+*^	81 (89%)	10 (11%)	0	0.11	
*Nbeal2*^*gps/gps*^	35 (51%)	19 (27%)	15 (22%)	0.71	1.898x10^-8^

* 3 slides per genotype, >20 megakaryocytes per slide

### Emperipolesis of neutrophils in bone marrow and spleen of *Nbeal2*^*gps/gps*^ mice

**Fig 4:**
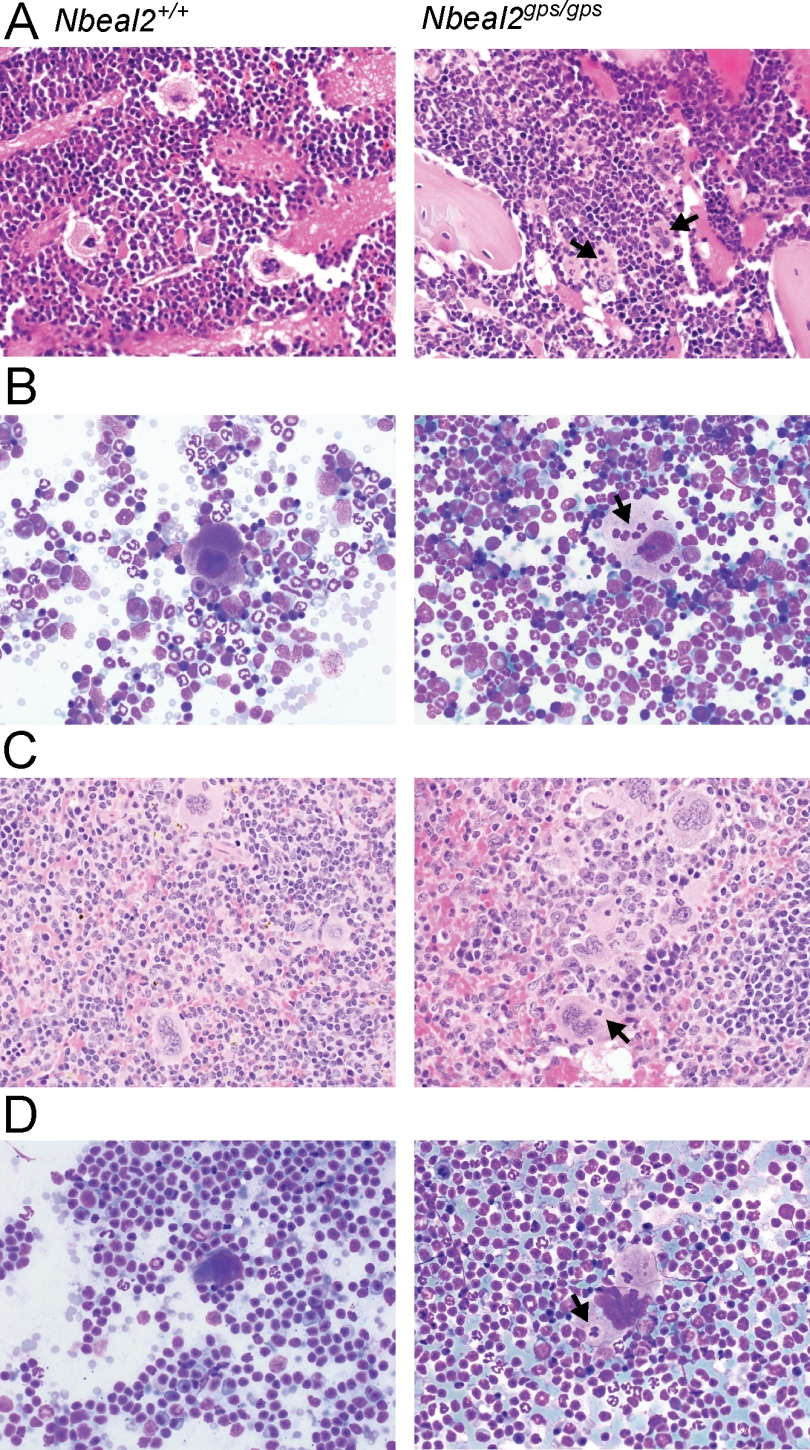
Emperipolesis of neutrophils in bone marrow and spleen of *NBEAL2* deficient mice. Increased emperiopolesis of neutrophils (black arrows) in *Nbeal2*^*gps/gps*^ mice compared to wildtype was observed in both histologic (A) and cytologic (B) preparations of bone marrow as well as spleen (B and D, respectively).

*Nbeal2*^*gps/gps*^ mice exhibit higher levels of megakaryocytic emperipolesis (the presence of an intact cell within the cytoplasm of another cell) in the bone marrow compared to wildtype mice, consistent with previously reported human and mouse GPS phenotypes [20, 21, 41–43]. Though emperipolesis is occasionally observed in megakaryocytes of wildtype mice (~11%), approximately half of the bone marrow megakaryocytes in *Nbeal2*^*gps/gps*^ mice exhibited some degree of emperipolesis (Fig 4A and 4B, Table 4, p = 1.9 x 10^−8^). Megakaryocytes containing more than one neutrophil were observed exclusively in the bone marrow of *Nbeal2*^*gps/gps*^ mice. Similarly, increased emperipolesis was observed in spleens of *Nbeal2*^*gps/gps*^ mice (Fig 4C and 4D). *Nbeal2*^*gps/gps*^ bone marrows demonstrated no defect in myeloid maturation though there appeared to be a mild increase in myeloid and megakaryocytic extramedullary hematopoiesis.

## Discussion

We report the identification and characterization of a spontaneous *Nbeal2* mutation in 129S1/SvImJ. Homozygosity for this 8 bp frameshift results in loss of NBEAL2 expression and phenotypic features characteristic of GPS in humans [20, 24]. These findings are also consistent with three other previous reports of *Nbeal2* deficient mice generated by gene targeting [21–23].

Though an initial cohort of *Nbeal2*^*gps/gps*^ mice (set 1) demonstrated differences in neutrophil counts and mean platelet volumes (Table 3) compared to previously reported mouse and human phenotypes, these features were not confirmed in the second cohort (mice backcrossed 5 additional generations into C57BL/6J). These data suggest that the differences observed in set 1 mice are due to either strain background effects [44] or loosely linked passenger mutations [45] that were removed by consecutive backcrossing. Additional confounding factors could include the difference in age between the two mouse cohorts. Comparison of the absolute neutrophil count (ANC) from the Hemavet analyzer to the ANC obtained by flow cytometry, demonstrates consistent overestimates on the Hemavet. This discrepancy could be secondary to limitations of the Hemavet system in discerning between neutrophils and monocytes. Similar results have been previously reported [46]. The quantification of ANC by flow cytometry should identify the population corresponding exclusively to neutrophils. In addition, the use of Trucount counting tubes has been well validated and offers an internal control with respect to sample preparation. The estimated coefficient of variation for our flow cytometry ANC assay using the Trucount beads is 3.48%. Thus, we consider the results obtained by FACS to more accurately represent the ANC.

Our data establish that the *Nbeal2*^*gps*^ allele is a spontaneous mutation that arose in the 129S1/SvImJ stock at the Jackson Laboratory in 2007 at F63. Though, we were unable to confirm the presence of the mutation in archival samples, this is likely due to the small number of archived samples available. Published rates of spontaneous mutations in mice range from 10^−5^ to 10^−6^ per locus per gamete on the basis of specific locus testing with visible phenotypes [47]. More recently, whole genome sequencing and pedigree analyses have estimated a mutation rate of 5.4 x 10^−9^ per base/ per generation in wildtype laboratory mice [15], which is roughly 28 mutations, genome wide per generation / diploid genome. New mutations have a 25% chance of becoming fixed in an inbred population, assuming random segregation in the absence of selection [7]. We performed exome sequencing on a single 129S1/SvImJ trio (F63pF64 and F63pF65) and did not find a *de novo*, coding SNV or small INDEL (see Materials and Methods). This is consistent with previously published mutation rates (given a ~50 Mb exome, at 10X minimum coverage where the likelihood of detecting a germ line *de novo*, exonic mutation is ~5% in any individual).

The *Nbeal2*^*gps*^ allele was identified via whole exome sequencing of the progeny of an ENU treated mouse. While next generation sequencing approaches have high utility for mapping both spontaneous [48] as well as chemically induced *de novo* variants [49], the origin of a single nucleotide variant cannot be established from sequencing data alone. While ENU-induced mutations are certainly the most common in an ENU colony, spontaneous mutations are also present at predictable frequencies and unlike ENU mutations, spontaneous mutations are not limited to SNVs and can include structural alterations (copy number variants and rearrangements). Therefore, while infrequent, it is not surprising that spontaneous mutations with relevant phenotypes have been recovered in ENU screens [50].

Ultimately, the origin of causative mutations (ENU or spontaneous) can be established through additional genotyping of the ENU pedigree, assuming breeding records and samples have been carefully maintained and archived. Generally, strong dominant phenotypes due to *de novo* variants are easily detected in mouse colonies; however, mild dominant phenotypes or recessive phenotypes may go unnoticed depending on the breeding paradigm. For these reasons, it is important to adhere to published guidelines on mouse colony management and genetic quality control monitoring [51]. In the case of the *Nbeal2*^gps^ allele, the platelet defect had no impact on survival of *F5*^L/L^ *Tfpi*^+/-^ mice and we were able to identify the variant only due to next generation sequencing.

## Supporting Information

S1 Fig Schematic overview of the *Nbeal2* genotyping primers. (PNG)

S2 Fig *MF5L*6 suppressor line pedigree. Only progeny mice with the *F5*^*L/L*^ *Tfpi*^+/-^ genotype and unaffected parents are shown in the pedigree. Black boxes highlight the mice subjected to whole exome sequencing. The red box highlights mouse 67339 that was used for *Nbeal2*^*gps*^ allele outcrossing and line establishment.
(PDF)

S3 Fig Genotyping of 129S1/SvImJ archived samples from The Jackson Laboratory. Two different mice (A91, A92) purchased from The Jackson Laboratory had *Nbeal2*^*gps/+*^ progeny (red). One of these progeny (asterisk) was the sire of the female used to build the ENU suppressor line (A). All genotyped 129S1/SvImJ (JR# 002448) mice were wildtype at the *Nbeal2* locus, including the “Adam and Eve” founders of The Jackson Laboratory GSP 129S1/SvImJ stock (F60) [7] and two subsequent generations of cryopreserved embryo stock (F61, F63) (B). The *Nbeal2* deletion was also absent in two post-GSP 129S1/SvImJ animals: whole exome sequencing data (F63pF67) from the Mouse Mutant Resource [32] and whole genome sequencing data (F63pF65) from the Sanger Mouse Genomes Project (C) [1].
(PDF)

S4 Fig Differential allelic expression of *Nbeal2* mRNA in *Nbeal2*^*gps/+*^ bone marrow, lung, and liver. Allelic expression was measured at every position in the Sanger sequenced RT-PCR product where the reference and deletion alleles had a different nucleotide. Dotted lines fill the gaps. In all tested tissues, the relative expression of *Nbeal2*^*gps*^ allele is lower than wildtype, set as 100% (A). Boxplot of all data points show on average ~65% reduction in expression of the deletion allele (B).
(PDF)

S5 Fig Western blot analysis for *NBEAL2* and beta Actin. Here we show full blots for the Western blot analysis. Areas surrounded by black boxes were displayed in Fig 1C.
(PDF)
