# Supplementary figures and images for: Spontaneous 8bp Deletion in Nbeal2 Recapitulates the Gray Platelet Syndrome in Mice
Source: PLoS One. 2016 Mar 7;11(3):e0150852. doi: 10.1371/journal.pone.0150852 (PMC4780761; doi:10.1371/journal.pone.0150852)

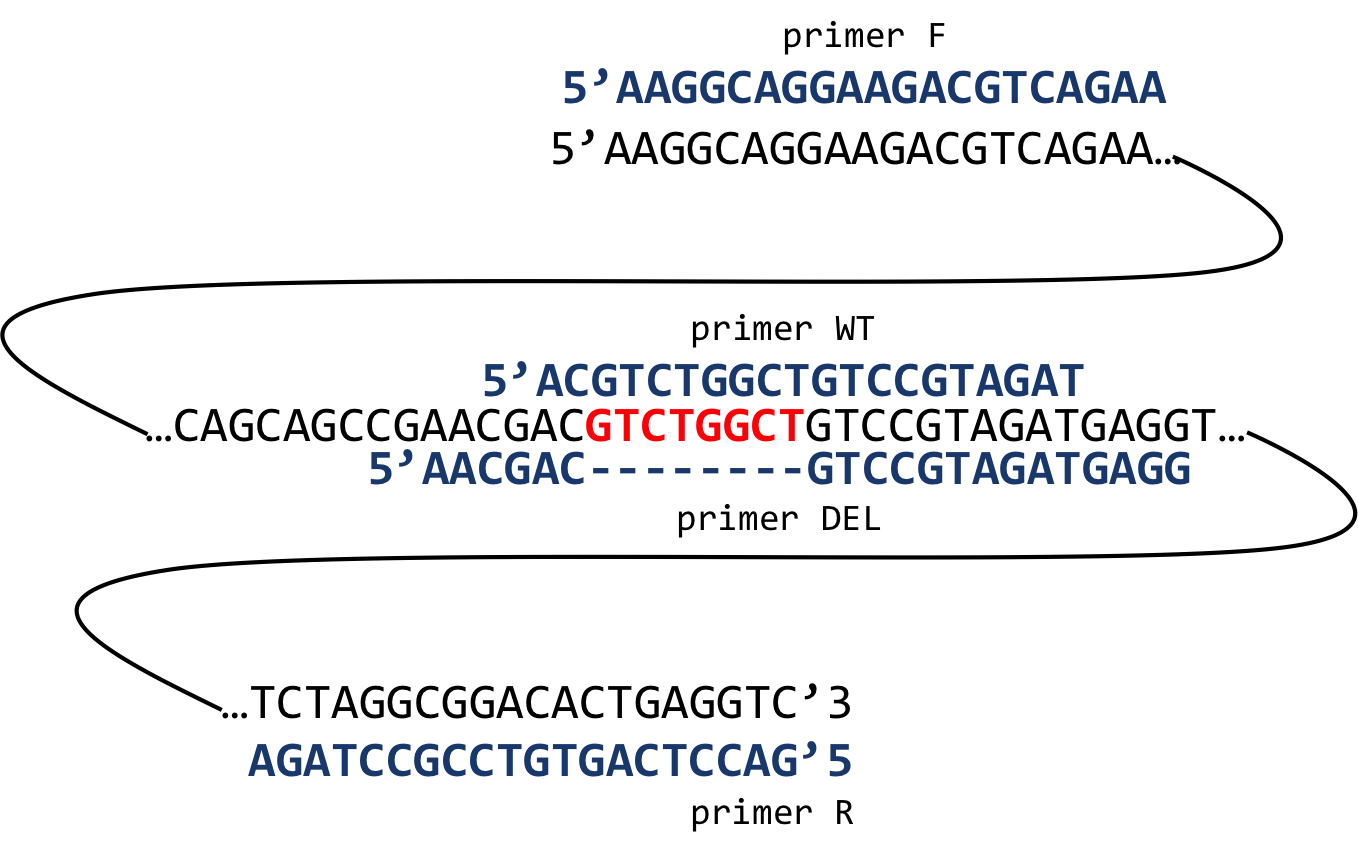

Supplement: S1 Fig — (PNG) [file pone.0150852.s001.png]

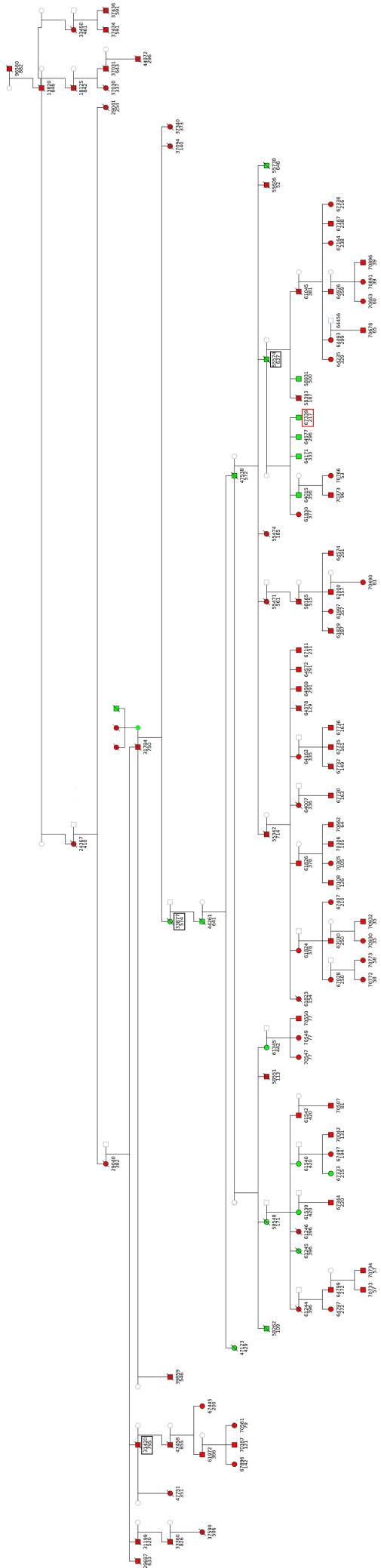

Supplement: S2 Fig — Only progeny mice with the F5L/L Tfpi+/- genotype and unaffected parents are shown in the pedigree. Black boxes highlight the mice subjected to whole exome sequencing. The red box highlights mouse 67339 that was used for Nbeal2gps allele outcrossing and line establishment. (PDF) [file pone.0150852.s002.pdf]

A

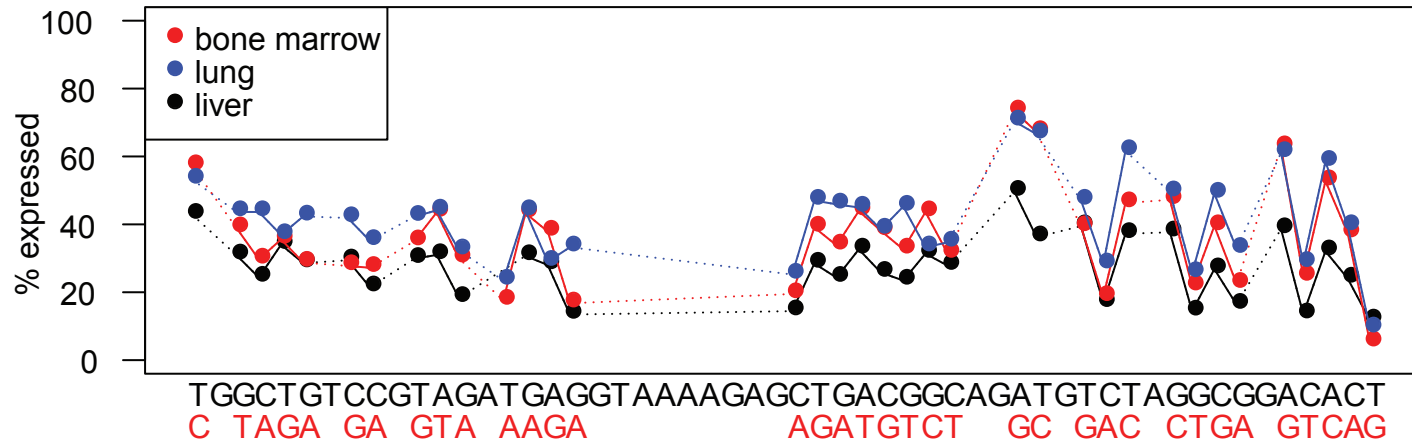

B

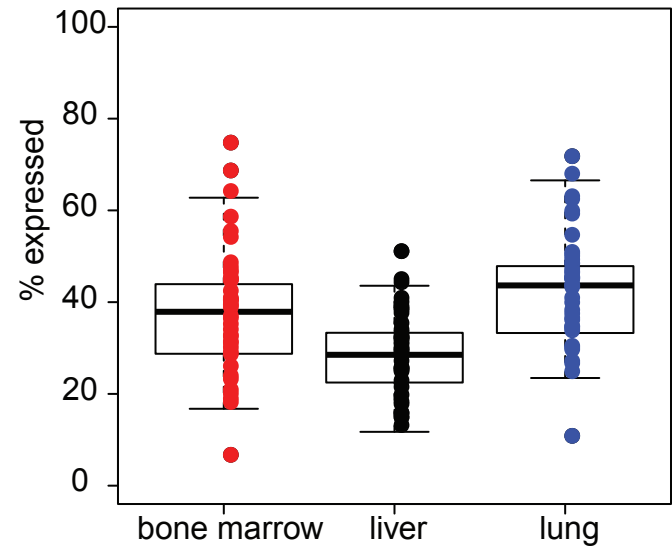

Supplement: S4 Fig — Allelic expression was measured at every position in the Sanger sequenced RT-PCR product where the reference and deletion alleles had a different nucleotide. Dotted lines fill the gaps. In all tested tissues, the relative expression of Nbeal2gps allele is lower than wildtype, set as 100% (A). Boxplot of all data points show on average ~65% reduction in expression of the deletion allele (B). (PDF) [file pone.0150852.s004.pdf]

A

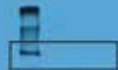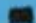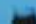

B

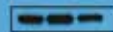

Supplement: S5 Fig — Here we show full blots for the Western blot analysis. Areas surrounded by black boxes were displayed in Fig 1C. (PDF) [file pone.0150852.s005.pdf]
