# Supplementary material for: Spontaneous 8bp Deletion in Nbeal2 Recapitulates the Gray Platelet Syndrome in Mice
Source: PLoS One. 2016 Mar 7;11(3):e0150852. doi: 10.1371/journal.pone.0150852 (PMC4780761; doi:10.1371/journal.pone.0150852)
Supplement: S3 Fig — Two different mice (A91, A92) purchased from The Jackson Laboratory had Nbeal2gps/+ progeny (red). One of these progeny (asterisk) was the sire of the female used to build the ENU suppressor line (A). All genotyped 129S1/SvImJ (JR# 002448) mice were wildtype at the Nbeal2 locus, including the “Adam and Eve” founders of The Jackson Laboratory GSP 129S1/SvImJ stock (F60) [7] and two subsequent generations of cryopreserved embryo stock (F61, F63) (B). The Nbeal2 deletion was also absent in two post-GSP 129S1/SvImJ animals: whole exome sequencing data (F63pF67) from the Mouse Mutant Resource [32] and whole genome sequencing data (F63pF65) from the Sanger Mouse Genomes Project (C) [1]. (PDF) [file pone.0150852.s003.pdf]

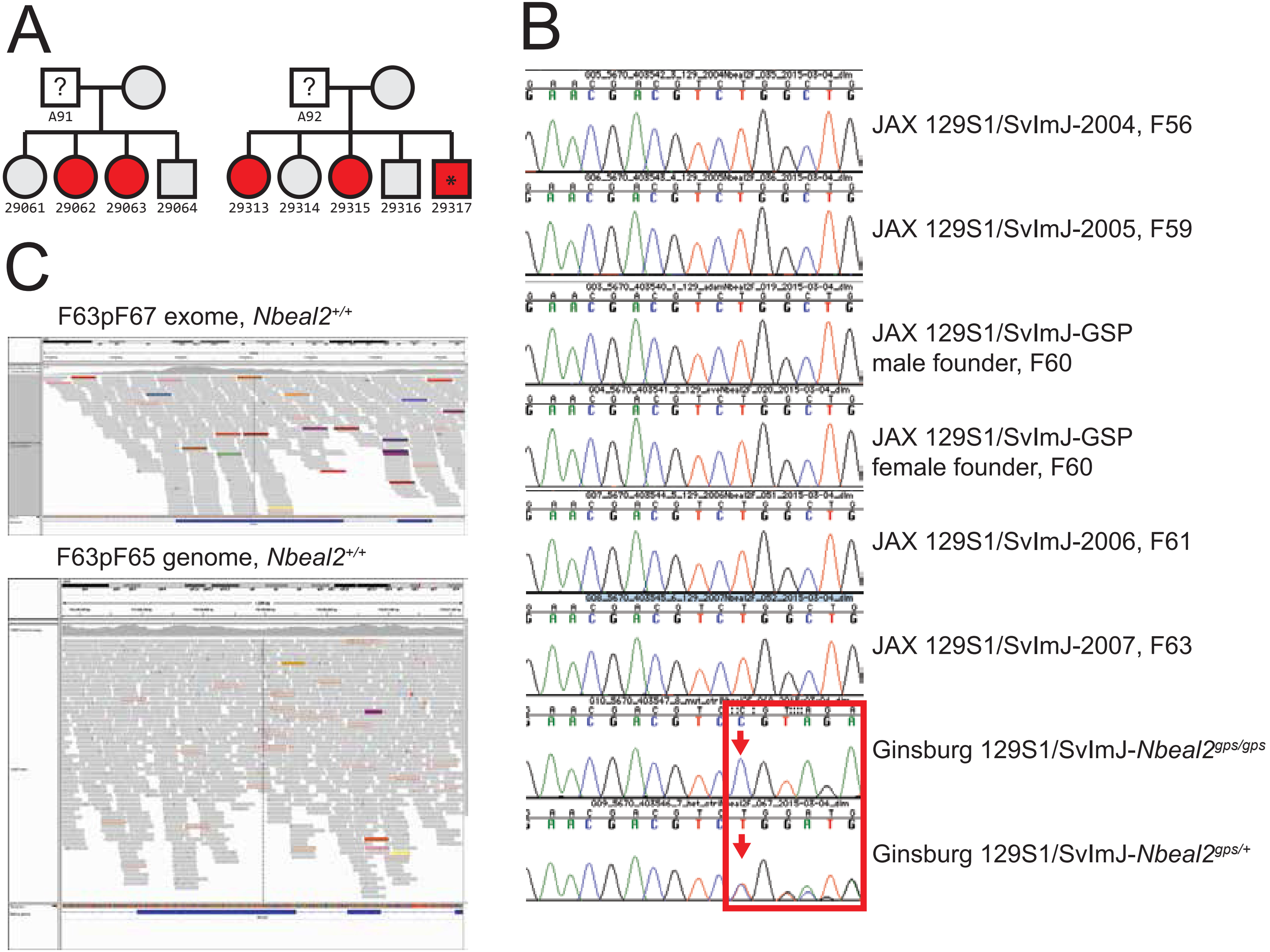
A
?
A91
?
A92
29061 29062 29063 29064
29313 29314 29315 29316 29317
*
B
JAX 129S1/SvImJ-2004, F56
JAX 129S1/SvImJ-2005, F59
JAX 129S1/SvImJ-GSP male founder, F60
JAX 129S1/SvImJ-GSP female founder, F60
JAX 129S1/SvImJ-2006, F61
JAX 129S1/SvImJ-2007, F63
Ginsburg 129S1/SvImJ-Nbeal2gps/gps
Ginsburg 129S1/SvImJ-Nbeal2gps/+
C
F63pF67 exome, Nbeal2+/+
F63pF65 genome, Nbeal2+/+
